# Coping with brain amyloid: genetic heterogeneity and cognitive resilience to Alzheimer’s pathophysiology

**DOI:** 10.1186/s40478-021-01154-1

**Published:** 2021-03-23

**Authors:** Vijay K. Ramanan, Timothy G. Lesnick, Scott A. Przybelski, Michael G. Heckman, David S. Knopman, Jonathan Graff-Radford, Val J. Lowe, Mary M. Machulda, Michelle M. Mielke, Clifford R. Jack, Ronald C. Petersen, Owen A. Ross, Prashanthi Vemuri, for the Alzheimer’s Disease Neuroimaging Initiative (ADNI)

**Affiliations:** 1 Department of Neurology, Mayo Clinic-Minnesota, 200 First Street SW, Rochester, MN 55905 USA; 2 Department of Health Sciences Research, Mayo Clinic-Minnesota, Rochester, MN 55905 USA; 3 Department of Radiology, Mayo Clinic-Minnesota, 200 First Street SW, Rochester, MN 55905 USA; 4 Division of Biomedical Statistics and Informatics, Mayo Clinic-Florida, Jacksonville, FL 32224 USA; 5 Department of Psychiatry and Psychology, Mayo Clinic-Minnesota, Rochester, MN 55905 USA; 6 Department of Neuroscience, Mayo Clinic-Florida, Jacksonville, FL 32224 USA; 7 Department of Clinical Genomics, Mayo Clinic-Florida, Jacksonville, FL 32224 USA

**Keywords:** Alzheimer’s, Amyloid, Cognitive decline, Genome-Wide Association Study, Resilience

## Abstract

Although abnormal accumulation of amyloid in the brain is an early biomarker of Alzheimer’s disease (AD), wide variation in cognitive trajectories during life can be seen in the setting of brain amyloidosis, ranging from maintenance of normal function to progression to dementia. It is widely presumed that cognitive resilience (i.e., coping) to amyloidosis may be influenced by environmental, lifestyle, and inherited factors, but relatively little in specifics is known about this architecture. Here, we leveraged multimodal longitudinal data from a large, population-based sample of older adults to discover genetic factors associated with differential cognitive resilience to brain amyloidosis determined by positron emission tomography (PET). Among amyloid-PET positive older adults, the AD risk allele *APOE* ɛ4 was associated with worse longitudinal memory trajectories as expected, and was thus covaried in the main analyses. Through a genome-wide association study (GWAS), we uncovered a novel association with cognitive resilience on chromosome 8 at the *MTMR7/CNOT7/ZDHHC2/VPS37A* locus (*p* = 4.66 × 10^–8^, β = 0.23), and demonstrated replication in an independent cohort. Post-hoc analyses confirmed this association as specific to the setting of elevated amyloid burden and not explained by differences in tau deposition or cerebrovascular disease. Complementary gene-based analyses and publically available functional data suggested that the causative variant at this locus may tag *CNOT7* (CCR4-NOT Transcription Complex Subunit 7), a gene linked to synaptic plasticity and hippocampal-dependent learning and memory. Pathways related to cell adhesion and immune system activation displayed enrichment of association in the GWAS. Our findings, resulting from a unique study design, support the hypothesis that genetic heterogeneity is one of the factors that explains differential cognitive resilience to brain amyloidosis. Further characterization of the underlying biological mechanisms influencing cognitive resilience may facilitate improved prognostic counseling, therapeutic application, and trial enrollment in AD.

## Introduction

Observational studies support a dynamic model of Alzheimer’s disease (AD), in which amyloidosis is an early event that is eventually followed by other biomarker abnormalities and clinical impairment [24, 27]. However, a considerable fraction of older adults display abnormal accumulation of amyloid in the absence of overt cognitive impairment, with the frequency of this finding modified by age, *APOE* (apolipoprotein E) ɛ4 status, and sex, among other factors [7, 28, 30]. This observation highlights the importance of elucidating the underlying biological mechanisms of resilience to (i.e., coping with) amyloid pathology within frameworks attempting to forecast cognitive outcomes in AD [3].

It is widely presumed that resilience may be influenced by the environment and lifestyle [41] in addition to inherited factors [23]. The recent discovery of the *APOE* (apolipoprotein E) Christchurch mutation as protective in the face of high amyloid burden and an AD-causative *PSEN1* (presenilin 1) mutation represents a germane exemplar for this concept [1]. However, there is a relative paucity of literature on genetic resilience factors specific to AD. Analyses of general cognitive decline in the wider setting of older adults have most consistently implicated variants in the *APOE* region on chromosome 19 [13–15, 70]. A few studies have examined putative downstream effects in the setting of AD pathology for specific candidate loci, including the *APOE* ɛ4 [46], *KL* (klotho) VS [6], and *BDNF* (brain-derived neurotrophic factor) Val66Met [19] alleles, among others. However, much of the presumed genetic architecture underlying resilience to AD pathology is still unaccounted for by known candidate genes.

The validation and expansion of positron emission tomography (PET) imaging biomarkers creates the opportunity to noninvasively assess in vivo AD pathology in large samples conducive for discovery-oriented genetic analyses of resilience. In this study, we hypothesized that novel gene variants and biological pathways would be associated with differential cognitive resilience to amyloidosis. We tested this hypothesis by conducting a genome-wide association study (GWAS) of longitudinal memory performance in a large, population-based sample of amyloid-PET positive older adults.

## Methods

### Selection of participants

The discovery cohort in this study was drawn from the Mayo Clinic Study of Aging (MCSA), a population-based prospective study of older adults residing in Olmsted County, Minnesota [49, 54]. Individuals were identified for recruitment using the Rochester Epidemiology Project (REP) medical records linkage system [56, 61]. Enrollment began in 2004 for individuals 70–89 years of age, and the study was subsequently extended to include those aged 50 and older (2012) and 30 and older (2015). Clinical data through questionnaires and in-person history, neuropsychological assessment, multimodal neuroimaging, and laboratory tests were assessed at selected visits based on study protocols. Clinical diagnoses were made by a consensus panel, incorporating all available information. All study protocols were approved by the Mayo Clinic and Olmsted Medical Center Institutional Review Boards. Written informed consent was obtained from all participants or their surrogates.

The replication cohort analyzed separately in this study was drawn from the Alzheimer’s Disease Neuroimaging Initiative (ADNI), a longitudinal multicenter study launched in 2004 as a public–private partnership [63, 68]. The goal of the ADNI is to facilitate development of clinical, imaging, genetic, and biochemical biomarkers for the early detection and tracking of AD. Individuals 55–90 years of age were recruited from over 50 sites across the United States and Canada, and initially followed at 6–12-month intervals for 2–3 years. Subsequent study phases have extended follow-up for existing participants and have enrolled additional individuals. All participants provided written informed consent and study protocols were approved by each site’s institutional review board. Further information about the ADNI can be found at http://adni.loni.usc.edu/.

Inclusion criteria for this study included the following: age 50 years or older, the presence of amyloid positivity by PET, at least 2 subsequent time points with neuropsychological assessment data, and genome-wide single nucleotide polymorphism (SNP) genotype data. This resulted in 546 individuals in the MCSA discovery cohort and 545 individuals in the ADNI replication cohort. A separate, non-overlapping group of 953 amyloid PET negative individuals from the MCSA was analyzed post-hoc for comparison.

### Demographic and clinical data

Age at the time of neuroimaging, sex, and years of education were ascertained for each patient. In the discovery cohort, a measure of cerebrovascular disease risk (CMC) was ascertained from health care records as a summation of the presence or absence of hypertension, hyperlipidemia, cardiac arrhythmias, coronary artery disease, congestive heart failure, diabetes, and stroke [67].

### Longitudinal cognitive data

For each applicable participant visit in the MCSA discovery cohort, a composite memory domain z-score was generated as described previously [34, 55], based on the delayed recall tasks of the Wechsler Memory Scale-Revised Logical Memory II, Wechsler Memory Scale-Revised Visual Reproduction II, and Auditory Verbal Learning Test from cognitively unimpaired participants aged 50 and older and weighted back to the Olmsted County population. For each subject, linear regression on these memory z-scores was used to generate a subject-specific intercept and slope. The variability in slope across all of the subjects captures the variability in longitudinal trajectories of memory functioning across all of the subjects, and was used as our primary outcome measure. The intercept for each subject was used as a covariate in analyses, representing an estimate of early-age. For the ADNI replication cohort, the 13-item Alzheimer's Disease Assessment Scale-Cognitive (ADAS-Cog) Subscale was used as the analogous measure of interest based on previous work employing longitudinal cognitive data in ADNI [2, 39]. Linear regression was similarly used to generate a subject-specific intercept and slope for each participant based on longitudinal ADAS-Cog scores for the replication cohort, with the variability in slope across all subjects again used as the primary outcome.

### Genetic data

Peripheral blood samples were acquired at the baseline visit for 1783 MCSA participants. Genomic DNA extracted from these samples was used for genotyping of 658,805 SNPs via the Illumina Infinium Global Screening Array-24 v2.0. Standard SNP-level quality control (QC) filters were applied using PLINK version 1.9 [9, 51], including call rate ≥ 95%, Hardy–Weinberg Equilibrium *p* ≥ 1 × 10^–5^, and minor allele frequency (MAF) ≥ 1%. Subject-level QC filters included call rate ≥ 98%, sex checks versus clinical data, Caucasian ancestry determined through STRUCTURE version 2.3.4, and ensuring no cryptic first- or second-degree relatedness (PLINK identity by descent PI_HAT < 0.25). Following these procedures, GWAS array data passing QC was available for 506,136 SNPs and 1727 MCSA participants. *APOE* ɛ2/ɛ3/ɛ4 allele status determined through the GWAS array (via genotyping of rs429358 and rs7412) displayed 100% concordance with results from standard restriction digest methods [20]. As a conservative measure to account for any potential confounding effects of population stratification, principal component analysis of the genotype data with SNPRelate [71] was used to generate eigenvectors for use as covariates. Genome-wide imputation was performed with the Michigan Imputation Server [12] using Minimac version 4-–1.0.2 and the Haplotype Reference Consortium reference panel [43]. Following additional post-imputation QC filters including SNP call rate ≥ 95%, sample call rate ≥ 98%, Hardy–Weinberg Equilibrium *p* ≥ 1 × 10^–6^, MAF ≥ 1%, stringent imputation quality measure (*r*^2^) ≥ 0.8, and removal of SNPs with no or duplicate identifying rs number, data was available for 6,153,814 SNPs and 1727 MCSA participants. For this study, to ensure appropriate power for analyzed variants given the sample size in the discovery cohort, an additional MAF filter of 5% was applied, leaving 4,456,454 SNPs for analysis in the GWAS.

ADNI-1, ADNI-GO, and ADNI-2 participants were genotyped on one of three Illumina GWAS arrays as described previously [58]. Processed, post-QC genotype data files were downloaded from the ADNI database (http://adni.loni.usc.edu). Imputation was performed within groups based on the genotyping array utilized, and then the independently imputed datasets were merged using PLINK. Imputation methods and post-imputation QC were performed as in the MCSA dataset and resulted in 5,599,642 SNPs for 1662 unique ADNI participants. In the merged sample, a total of 18 individuals (representing 6 pairs and 2 trios) were found to have cryptic relatedness (PI_HAT ≥ 0.25) based on identity by descent analysis using common (MAF ≥ 5%) SNPs roughly pruned for LD (*r*^2^ < 0.6). After one individual from each pair or trio was randomly selected for retention, data was available for analysis for 1652 unique ADNI participants. As with the MCSA dataset, the first 5 principal component eigenvectors were used as covariates in genetic analyses.

### Neuroimaging data

In the MCSA, amyloid PET and tau PET scans were acquired and analyzed using an in-house fully-automated image processing pipeline as described in detail elsewhere [26]. Amyloid PET imaging was performed with Pittsburgh compound B (PiB) [33] and tau PET was performed with AV-1451 (^18^F-flortaucipir), synthesized on site with precursor supplied by Avid Radiopharmaceuticals [42]. Standardized uptake value ratio (SUVR) measures for amyloid and tau PET were generated by normalizing median tracer uptake in target regions of interest (ROIs) to the cerebellar crus grey matter. The target amyloid PET measure was global cortical amyloid load, computed from the prefrontal, orbitofrontal, parietal, temporal, anterior cingulate, and posterior cingulate/precuneus ROIs. Amyloid PET positivity was defined by global cortical SUVR ≥ 1.48 as previously described [42]. The target tau PET measure was a meta-ROI computed from the entorhinal, amygdala, parahippocampal, fusiform, and inferior and middle temporal ROIs [26]. Tau PET positivity was defined by meta-ROI SUVR ≥ 1.25 as previously described [25]. Entorhinal cortex tau PET burden was also assessed.

MRI for MCSA participants was performed on 3T MRI systems (General Electric Healthcare, Waukesha, WI). Target MRI biomarkers of neurodegeneration were derived using FreeSurfer version 5.3 and included hippocampal volume and the cortical thickness in an AD-signature meta-ROI comprised of the entorhinal cortex and inferior and middle temporal and fusiform gyri [59]. Target MRI biomarkers of cerebrovascular disease included white matter hyperintensities (WMHs) from FLAIR MRI [17] and fractional anisotropy (FA) of the genu of the corpus callosum from diffusion tensor imaging (DTI) MRI [66].

In the ADNI, amyloid PET was performed with ^18^F-florbetapir using acquisition and processing protocols as described at http://www.adni-info.org. Amyloid PET summary measures generated at the University of California, Berkeley [29] were downloaded from the ADNI database (http://adni.loni.usc.edu). The target amyloid PET measure was global cortical amyloid load, assessed from FreeSurfer-defined regions of interest including the anterior and posterior cingulate, frontal, lateral parietal, and lateral temporal cortices, and normalized to a whole cerebellum reference region [39]. Amyloid PET positivity was defined by global SUVR ≥ 1.11, as previously described [38].

### Statistical analysis

Genetic association tests with the memory slope phenotype were performed using PLINK version 1.9. Prior to the genome-wide scan in the discovery dataset, the *APOE* ɛ4 and ɛ2 alleles were analyzed for association signal given their well-validated relationship with AD and age-related cognitive trajectories. These candidate analyses and the GWAS were conducted on the discovery sample using linear regression under an additive genetic model and including age at the time of neuroimaging, sex, years of education, memory domain intercept (as a measure of premorbid performance), and the first 5 genetic principal component eigenvectors as covariates. For the GWAS, *APOE* ɛ4 status (presence versus absence) was also included as a covariate to account for its demonstrated association. The standard conservative threshold for genome-wide significance (*p* < 5 × 10^–8^) was used in the GWAS [48], and SNPs exceeding this association threshold in the discovery cohort were analyzed for confirmatory signal (*p* < 0.05) in the replication cohort. Effect sizes were denoted by standardized beta coefficients. Manhattan and Q-Q plots were generated using Haploview version 4.2 [4], and regional Manhattan plots were generated using the web-based tool LocusZoom [50].

To accompany the SNP-level GWAS results, gene-based analyses were performed using H-MAGMA [60], which utilizes functional genomics evidence via tissue-specific Hi-C chromatin interactions to map SNPs to genes. We applied the purified human astrocyte Hi-C data for mapping of SNPs to genes in H-MAGMA, given the high expression of degenerative-disorder-associated genes in these cells [60]. Summary *p *values were generated for 50,777 Ensembl transcript IDs [22], representing 18,038 unique HGNC gene IDs, and accounting for SNP-level association statistics and gene size and density. Complementary pathway analyses were performed using the GWAS SNP-based summary statistics, GSA-SNP2 [69], and the Canonical Pathways collection version 7.2 from the Molecular Signatures Database [62]. For the pathway analyses, set size was restricted to 5–200 genes to limit the potential for size-influenced spurious associations, and the false discovery rate (FDR) was used to account for multiple comparisons [52].

Additional analyses to extend and complement the GWAS were performed on the MCSA discovery cohort with SPSS Statistics version 22.0 (IBM Corp., Armonk, NY) and SAS version 9.4 (SAS Institute Inc., Cary, NC). Post-hoc models including cycle number to account for potential learning effects were substantially unchanged from the primary results. Within the discovery sample, gene variant associations with PET and MRI biomarkers were assessed, including age (at scan), sex, *APOE* ɛ4 status, and genetic principal components as covariates. For volume and thickness measures from MRI (hippocampal volume, AD meta-ROI cortical thickness, and FLAIR WMH), total intracranial volume was also included as a covariate. Post-hoc analyses of SNP associations with the primary memory phenotype utilized a supramaximal threshold of global PiB SUVR ≥ 2.0 (based on previous work [35]) to sub-stratify the sample into individuals with extremely high amyloid burden versus those with abnormal amyloidosis below this threshold (1.48 ≤ global PiB SUVR < 2.0). Separate post-hoc analyses also stratified the sample based on tau PET positivity.

## Results

### Sample characteristics

The discovery cohort (Table 1) included 546 individuals aged 50 years or older and amyloid PET positive from the population-based MCSA. All subjects had a baseline visit and at least one clinical follow-up with complete neuropsychological assessment data. Median follow-up time was 4.2 years (range 1–13 years) and the median number of longitudinal visits was 4 (range 2–11). Most of the sample (85%) had a diagnosis of cognitively unimpaired at the initial imaging visit, and just over half of the sample (58%) did not carry the *APOE* ɛ4 allele. The replication cohort, used for targeted validation of top SNPs from the discovery sample GWAS, included 545 amyloid PET positive adults aged 55 years or older from the ADNI. An additional sample of 953 amyloid PET negative older adults from the MCSA was analyzed post-hoc for comparison.

**Table 1:** Sample characteristics

	MCSA (Discovery)	MCSA (Comparison)	ADNI (Replication)
	N = 546	N = 953	N = 545
	Amyloid positive	Amyloid negative	Amyloid positive
Age (years)	77.0 (7.7)	70.0 (9.7)	76.1 (7.5)
Sex	255 (47%) women	430 (45%) women	260 (48%) women
	291 (53%) men	523 (55%) men	285 (52%) men
Education (years)	14.6 (2.8)	14.9 (2.5)	15.9 (2.8)
Median visit number	4 (1.9)	5 (1.8)	4 (1.9)
*APOE* ɛ4 status	317 (58%) negative	756 (79%) negative	193 (35%) negative
	229 (42%) positive	197 (21%) positive	352 (65%) positive
Diagnosis^a^	458 (85%) CU	887 (93%) CU	103 (19%) CU
	78 (14%) MCI	62 (7%) MCI	223 (41%) MCI
	6 (1%) Dementia	1 (0%) Dementia	214 (40%) Dementia

Values displayed as mean (standard deviation) or number (percentage)

*CU* cognitively unimpaired, *MCI* mild cognitive impairment

^a^Consensus clinical diagnosis at the first PET/clinical visit; data unavailable for 4 individuals in the MCSA Discovery, 3 individuals in the MCSA Comparison, and 5 individuals in the ADNI replication samples

### Hypothesis-driven analyses of APOE alleles with cognitive resilience to amyloidosis

We first analyzed the *APOE* ɛ4 and ɛ2 alleles given the body of literature relating these to AD and cognitive decline. Age at the imaging visit, sex, years of education, regression-derived memory domain intercept (as a measure of early-life performance), and genetic principal component eigenvectors were included as covariates. As expected, *APOE* ɛ4 was associated with worse memory trajectory in the setting of amyloid PET positivity under both dominant (*p* = 0.005, standardized β = -0.12) and additive (*p* = 0.003, β = -0.13) genetic models. *APOE* ɛ2 was present in 11% of the sample and was not associated with memory trajectory under additive (*p* = 0.31, β = 0.04) or dominant (*p* = 0.28, β = 0.05) models. Based on the validated association of the *APOE* ɛ4 allele with the primary outcome and to prioritize discovery of novel and independent SNP associations, *APOE* ɛ4 status (present versus absent) was included as an additional covariate in the GWAS.

### GWAS identifies novel association on chromosome 8 with cognitive resilience to amyloidosis

**Fig. 1:**
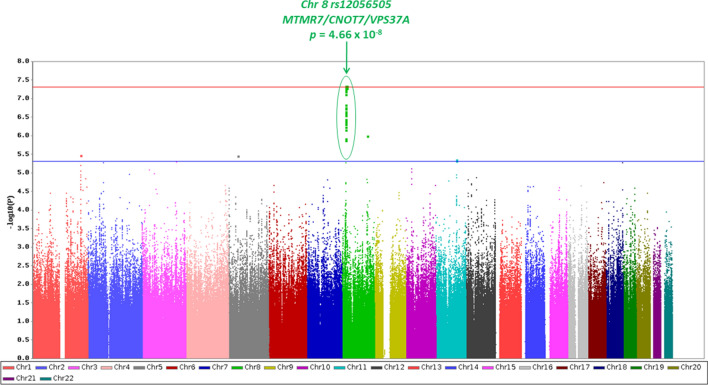
Manhattan plot for the GWAS of cognitive resilience to amyloidosis. The Manhattan plot displays observed −log_10_ *p *values (y-axis) for all single nucleotide polymorphisms (SNPs) tested in the GWAS of longitudinal cognitive trajectory in the setting of amyloid PET positivity. Age, sex, years of education, memory domain intercept (as a measure of premorbid performance), genetic principal components, and *APOE* ɛ4 status (presence versus absence) were included as covariates. An additive genetic model was utilized, along with a minor allele frequency filter of 5%. A genome-wide significant association (*p* < 5 × 10^–8^; red line) with cognitive resilience to amyloidosis was identified on chromosome 8. Suggestive associations (*p* < 5 × 10^–6^; blue line) were identified on additional chromosomes. Plot created using Haploview

**Fig. 2:**
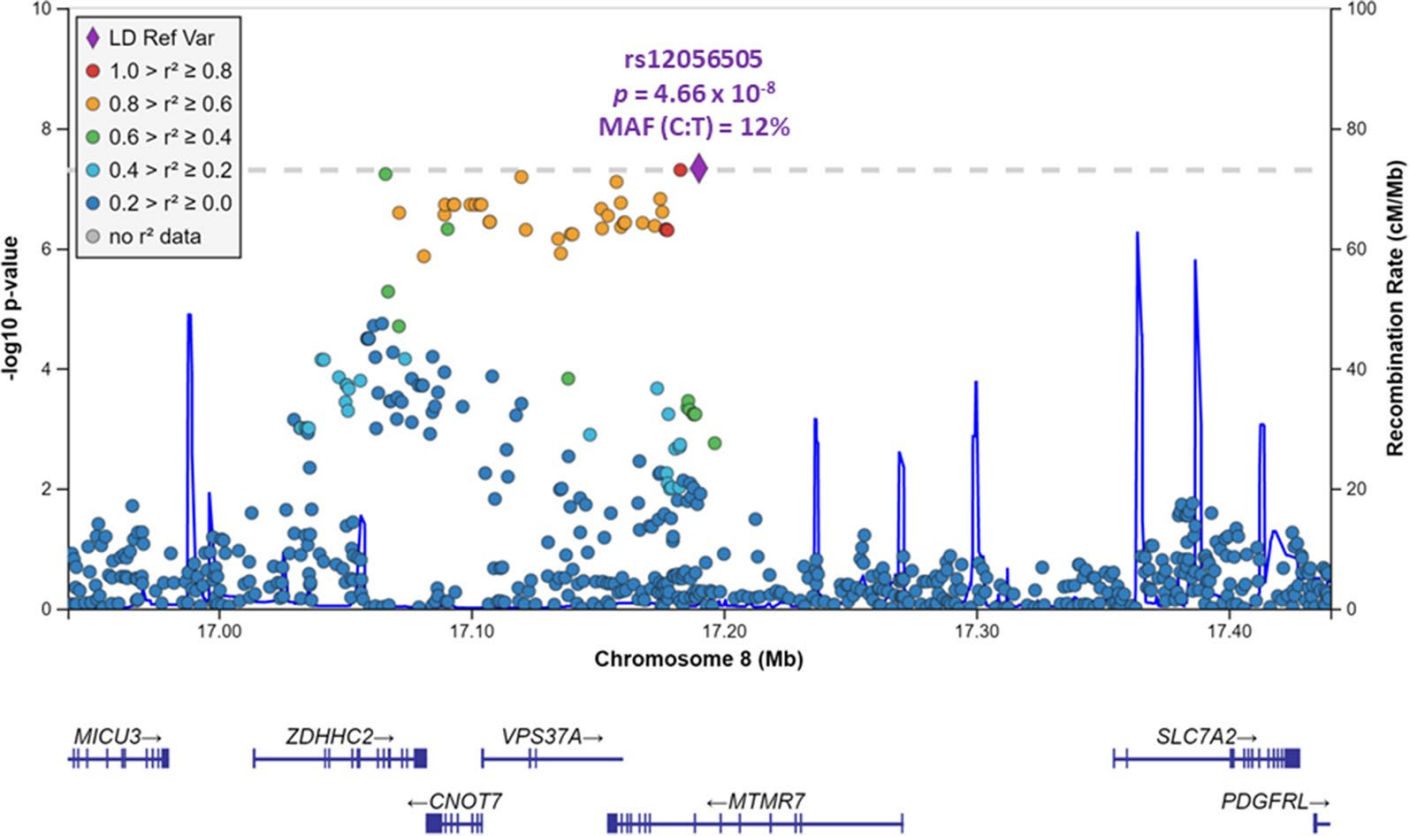
Regional Manhattan plot for the GWAS hit on chromosome 8. Regional association data are displayed for the genome-wide significant hit on chromosome 8. The top association signal in this region was for rs12056505 (denoted by the purple diamond), an intronic variant in the vicinity of *MTMR7* (myotubularin-related protein 7), *VPS37A* (vacuolar protein sorting-associated protein 37A), and *CNOT7* (CCR4-NOT transcription complex subunit 7). All variants within a 500 kb region surrounding the index SNP are plotted based on their association − log_10_ *p *values, NCBI build 37 genomic position, and recombination rates calculated from the 1000 Genomes Project reference data. The color scale of *r*^2^ values is used to label SNPs based on their degree of linkage disequilibrium with the index SNP. Genes in the region are labeled below the plots, with arrows denoting 5′-to-3′ orientation. Plot created using the LocusZoom software suite

There was no evidence of spurious inflation of association statistics in the GWAS (λ = 1.01). A novel genome-wide significant association (*p* < 5 × 10^–8^) with cognitive resilience in the setting of amyloid PET positivity was identified on chromosome 8 (Fig. 1). The top associated SNP in this region (Fig. 2) was rs12056505 (*p* = 4.66 × 10^–8^, β = 0.23), an intronic variant in *MTMR7* (myotubularin-related protein 7) that overlaps with the 3′ untranslated region of *VPS37A* (vacuolar protein sorting-associated protein 37A) and is nearby *CNOT7* (CCR4-NOT transcription complex subunit 7). Suggestive associations (*p* < 5 × 10^–6^) were identified at additional loci, including *CSMD3* (CUB and sushi multiple domains 3) which has been proposed as a regulator of dendrite development in hippocampal neurons [45], and *OXCT1* (3-oxoacid CoA-transferase 1) which resides in a region found to have differential DNA methylation in dementia cases versus controls [18] (Table 2).

**Table 2:** Top independent loci from the genome-wide association study

CHR	SNP	Gene	Minor/major allele	MAF	Std. beta	*p *value
8	**rs12056505**	***MTMR7***	**C/T**	**0.120**	**0.23**	**4.66 × 10**^**–8**^
8	rs2251983	*CSMD3*	A/C	0.454	− 0.20	1.00 × 10^–6^
1	rs10863241	Intergenic	A/G	0.458	0.20	3.38 × 10^–6^
5	rs17291699	*OXCT1*	C/T	0.065	− 0.19	3.56 × 10^–6^
11	rs11020813	*LINC01171*	C/A	0.101	0.19	4.51 × 10^–6^

*CHR* chromosome, *SNP* single nucleotide polymorphism, *MAF* minor allele frequency

Bold text indicates genome-wide significant association (*p* < 5 × 10^–8^)

### Association of Chromosome 8 rs12056505 with resilience is specific to the amyloid-positive setting and is validated in an independent sample

The GWAS hit for rs12056505 implicated a common variant, with MAF = 12% for the C allele. This association was dose-dependent, with more positive memory trajectories observed for rs12056505-CC (mean slope = 0.18, standard error = 0.09, n = 8) and rs12056505-CT (mean slope = 0.02, standard error = 0.02, n = 131) individuals than for rs12056505-TT individuals (mean slope = -0.09, standard error = 0.02, n = 407). The association of rs12056505-C with cognitive resilience was specific to the amyloid-positive setting, as no corresponding signal was observed in a comparison cohort of amyloid-negative individuals from the MCSA (*p* = 0.78, β = 0.01). In analyses of an independent cohort of 545 amyloid-positive individuals from the ADNI, we identified modest evidence of replication of the association of rs12056505 with cognitive resilience to amyloidosis (*p* = 0.04, β = 0.09).

### Post-hoc analyses in the MCSA provide context for the effect of rs12056505 on resilience

For deeper characterization of the GWAS hit, we further examined rs12056505 against relevant imaging biomarkers (Table 3). In the MCSA discovery sample, rs12056505-C was weakly associated with lower global amyloid PET burden, but its association with cognitive trajectory remained robust after additionally covarying for amyloid burden in the main model (*p* = 9.95 × 10^–8^, β = 0.22). In addition, when the sample was stratified into “extreme amyloid” (global PiB SUVR ≥ 2.0, n = 163) versus “high amyloid” (1.48 ≤ global PiB SUVR < 2.0, n = 383) groups based on prior work [35], the effect sizes for the association of rs12056505 with resilience were not substantially different (β = 0.26 in extreme amyloid group versus β = 0.22 in high amyloid group). Collectively, these results suggest that the association of rs12056505 with resilience was not purely mediated by differences in amyloid load.

**Table 3:** Associations of the top GWAS Hit with neuroimaging biomarkers in the MCSA

Biomarker	Chr 8 rs12056505
Global amyloid PET	*p* = 0.03
*N* = 546	β = − 0.09
AD-signature tau PET	*p* = 0.12
*N* = 106	β = − 0.15
Entorhinal cortex tau PET	*p* = 0.09
*N* = 106	β = − 0.17
White matter hyperintensities	*p* = 0.11
*N* = 369	β = − 0.07
Corpus callosum genu fractional anisotropy	*p* = 0.83
*N* = 420	β = 0.01
Hippocampal volume	*p* = 0.02
*N* = 529	β = 0.08
AD-signature cortical thickness	*p* = 0.01
*N* = 531	β = 0.10

In addition, rs12056505 was not significantly associated with MRI WMH burden or DTI-based FA of the corpus callosum genu, both measures of vascular brain injury. Its association with cognitive resilience was also unchanged after additionally covarying for an index score of cerebrovascular disease risk factors [67]. Altogether, these results indicate that the association of rs12056505 with resilience was not mediated by better vascular brain health.

This SNP was also nominally associated with higher AD-signature cortical thickness and hippocampal volume, and among the small proportion of the sample with corresponding tau PET (106/546 = 19%), the association of rs12056505 with lower entorhinal cortex tau PET burden was marginally nonsignificant. However, the effect size for rs12056505 remained strong (β = 0.35) even after additionally covarying for tau PET burden. Among the 34 individuals who were A + /T + (abnormally elevated amyloid and tau by PET), the effect size for rs12056505 (β = 0.26) was comparable to that of the full sample, indicating that its protective association was not restricted to individuals without substantial AD biomarker abnormalities. In addition, for rs12056505-C carriers compared to non-carriers there were no differences in sex (49% men versus 55% men, *p* = 0.23) or years of education (14.5 years versus 14.7 years, *p* = 0.46), indicating that rs12056505 genotype was not a proxy for these factors.

### Gene- and pathway-based associations with resilience to amyloidosis

To augment the SNP-level GWAS results, we used gene- and pathway-based analyses to assess for broader patterns of association signal across biologically-relevant combinations of variants. Utilizing H-MAGMA [60], the top gene-based association was for *CNOT7* (*p* = 1.65 × 10^–7^), which was genome-wide significant based on a stringent Bonferroni correction (*p* < 0.05/50,777 = 9.85 × 10^–7^). Additional strong gene-based associations are listed in Table 4. Complementary pathway analysis using GSA-SNP2 [69] revealed enrichment of association with resilience for pathways related to integrin-related cell adhesion, TCF (T-cell factor) signaling related to the Wnt/β-catenin pathway, and IFN-γ (interferon gamma) signaling for immune activation, including two pathway-level associations significant after FDR correction (Table 5).

**Table 4:** Top HMAGMA gene-based associations in the GWAS of resilience to amyloidosis

Gene [Chromosome]	Number of SNPs	*p *value	Function
***CNOT7 [8]***	**15**	**1.65 × 10**^**–7**^	**Immune response**
*C11ORF97* [11]	4	2.59 × 10^–6^	Unknown
*VPS37A* [8]	11	3.31 × 10^–6^	Endosomal sorting/trafficking
*ZDHHC2* [8]	40	8.20 × 10^–6^	Cell adhesion; synaptic plasticity
*RCC1L* [7]	20	2.47 × 10^–5^	Guanine nucleotide exchange factor
*LINC02449* [12]	35	6.34 × 10^–5^	Unknown
*MYOCOS* [1]	29	7.23 × 10^–5^	Unknown
*RAD24* [8]	6	7.45 × 10^–5^	DNA repair/checkpoint pathways
*LINC00987* [12]	11	8.53 × 10^–5^	Unknown
*VSTM5* [11]	82	8.64 × 10^–5^	Cell adhesion; neuronal morphology

^*^Bold text indicates gene-based genome-wide significant association (*p* < 9.85 × 10^–7^)

**Table 5:** Top GSA-SNP2 pathway associations in the GWAS of resilience to amyloidosis

Pathway [Database]	Number of genes	*p* value	Function
**Integrin-2 pathway [PID]**	**26**	**4.17 × 10**^**–6**^	**Cell adhesion**
**Negative regulation of TCF-dependent signaling by WNT ligand antagonists [Reactome]**	**15**	**2.24 × 10**^**–5**^	**Immune response; signal transduction**
FCGR activation [Reactome]	11	7.14 × 10^–5^	Immune response
Keratinization [Reactome]	171	1.94 × 10^–4^	Cell adhesion
Adipogenesis [WikiPathways]	119	2.23 × 10^–4^	Lipid metabolism
Photodynamic therapy-induced unfolded protein response [WikiPathways]	26	5.94 × 10^–4^	Cellular stress response
SNARE interactions in vesicular transport [KEGG]	35	8.63 × 10^–4^	Neurotransmitter release
Formation of the cornified envelope [Reactome]	118	8.98 × 10^–4^	Keratinocyte cell barrier
Interferon gamma pathway [ST]	10	0.001	Immune response

^*^Bold text indicates FDR-corrected *p* < 0.05

### Candidate SNP associations with resilience in the GWAS

Within the GWAS, we additionally looked closely at a small set of a priori SNPs of interest, comprising AD risk loci and variants previously related to resilience phenotypes. Among 40 SNPs which previously displayed genome-wide significant associations with clinical AD diagnosis in large case–control studies through the IGAP consortium [36, 37], nominal associations (*p* < 0.05) were identified for three variants: the AD risk allele *BIN1* rs6733839-T was associated with worse memory trajectory (*p* = 0.01, β = -0.11); the AD protective allele *SORL1* rs11218343-C was associated with better memory trajectory (*p* = 0.04, β = 0.09); and the AD protective allele *MEF2C* rs190982-G was associated with worse memory trajectory (*p* = 0.03, β = -0.09). Although *TOMM40* rs2075650 displayed nominal association within a basic model not accounting for *APOE* ɛ4 (*p* = 0.001, β = -0.14), this association was attenuated in the GWAS which included *APOE* ɛ4 as a covariate (*p* = 0.07, β = -0.11). Replication signal was not identified for SNPs reported in prior work to have associations with resilience proxy measures, including *RAB10* (RAS oncogene family Rab10) rs142787485 (*p* = 0.50, β = 0.03) [53], *BDNF* rs6265 (*p* = 0.59, β = 0.02) [19], *KL* rs9536314 (*p* = 0.44, β = 0.03) [6], and *ATP8B1* (ATPase phospholipid transporting 8B1) rs2571244 (*p* = 0.28, β = 0.05) [16].

## Discussion

**Fig. 3:**
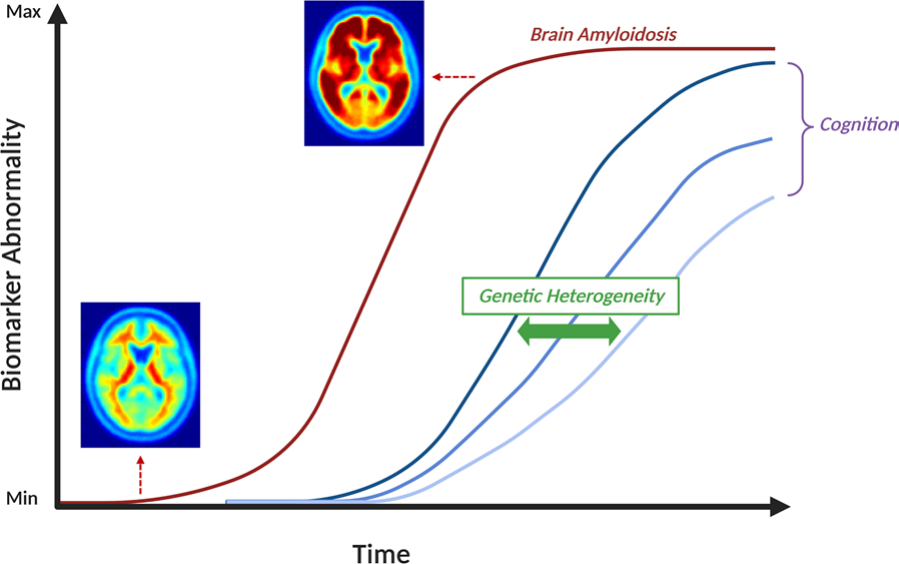
Conceptual model of genetic heterogeneity influencing cognitive resilience. A conceptual model displays the role of genetic heterogeneity in cognitive resilience to amyloidosis. Time is shown on the x-axis and points of maximum outcome abnormality (PET or cognitive functioning) are indicated by higher values along the y-axis. Sample cognitive trajectories, depicted by the blue curves, are shown in relation to the sigmoidal red curve depicting increasing brain PET amyloidosis over time. Genetic heterogeneity is shown as a modifier of cognitive resilience to amyloidosis, with more severe impairment (dark blue) related to a genetic risk profile and less impairment (light blue) related to a genetic protective profile. Figure adapted from Jack et al. [27] with the author’s permission

This study leveraged a large, population-based sample of amyloid PET positive older adults who had genome-wide SNP data and serial longitudinal cognitive assessments to understand genetic factors that contribute to cognitive resilience. Through this design, we discovered a novel association with cognitive resilience to amyloidosis for a locus on chromosome 8 which was specific to the amyloid positive setting and displayed replication in an independent cohort. We also identified biological pathways with enrichment of association, including a preponderance related to immune system activation. Our data support the hypothesis that genetic heterogeneity is one of the factors that explains differential cognitive resilience to brain amyloidosis (Fig. 3).

Resilience to brain amyloidosis is likely complex. It is well-understood that environmental/lifestyle factors, such as early- and late-life intellectual enrichment and cerebrovascular disease, will influence cognitive trajectories in older adults [41, 47, 64, 65]. Genetic factors are widely presumed to impact the degree of AD pathology, but relatively little of this architecture has been studied in the context of individuals with cognitive resilience in the setting of high levels of AD pathology. Further, the work so far at a population level has focused mainly on *APOE*. Case–control GWAS designs have been fruitful in identifying non-*APOE* risk variants for AD dementia, but may not be ideal to discover disease-specific resilience factors given the discrepancies between clinically diagnosed probable AD dementia versus biologically defined AD [5] as well as the heterogeneity amongst controls, some of whom may have extant AD pathophysiology while remaining non-demented at the time of study inclusion. Our approach using an easily interpretable and generalizable setting (amyloid positivity in a population-based sample of older adults) and integrating imaging biomarkers, genetics, and longitudinal clinical follow-up expands knowledge about heritable resilience factors in AD.

The top hit in our GWAS resided in *MTMR7*, which is ubiquitously expressed in the brain and contains a different SNP previously associated with susceptibility to Creutzfeldt-Jakob disease [57]. However, gene-based analyses of this region utilizing brain Hi-C for SNP-to-gene mapping suggested that the causative variant at this locus may instead tag *CNOT7*, a gene linked to synaptic plasticity and hippocampal-dependent learning and memory in model systems [44]. In addition, rs12056505 is a splicing quantitative trait locus (sQTL) for *CNOT7* in cultured fibroblasts (*p* = 1.5 × 10^–7^) [10] and may be an expression quantitative trait locus (eQTL) for *CNOT7* in brain cortex (*p* = 5.82 × 10^–3^) [22]. The nearby gene *VPS37A* has also been linked to hereditary spastic paraplegia [72] and belongs to a family of sorting proteins which may be important for tau clearance [40]. Our new findings call attention to this gene-rich region on chromosome 8 for further functional characterization, particularly given that top associated SNPs may not always represent the true functional variant at a locus.

**Fig. 4:**
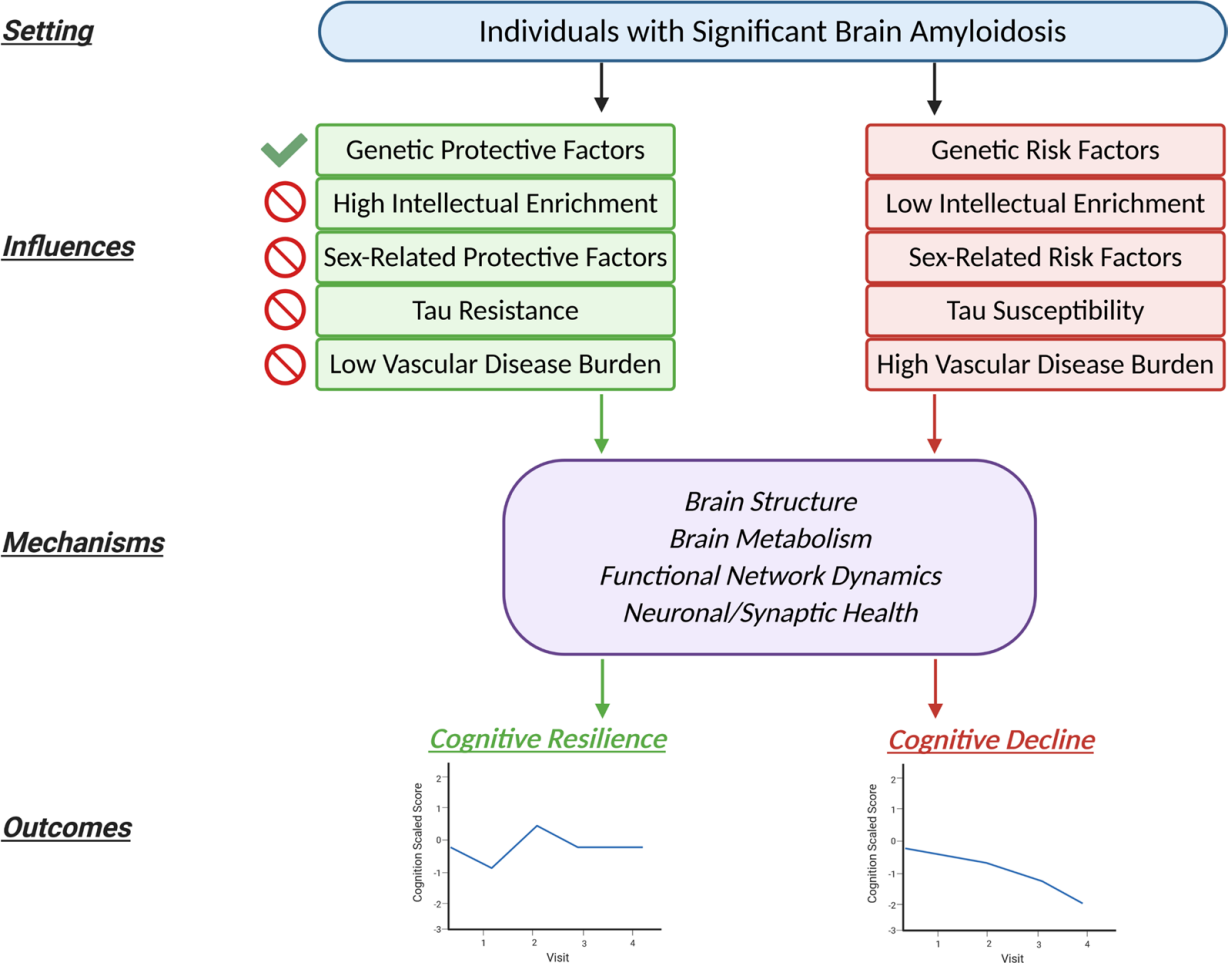
Experimental design for the discovery of genetic factors influencing cognitive resilience to amyloidosis. This study analyzed older individuals with significant brain amyloidosis and found evidence for genetic factors associated with cognitive resilience in that setting (green check mark). Alternative potential mediators for resilience were assessed, including intellectual enrichment, sex-related factors, resistance to tau accumulation, and cerebrovascular disease burden, and not explanatory for the genetic associations (red backslash icon). Our findings nominate new targets which warrant further study of the underlying molecular processes which impact brain structure and metabolism, functional network connectivity, and neuronal and synaptic health, to ultimately account for differential coping with amyloid pathology

It is still an open question whether the mechanisms underlying resilience are fundamentally mediated through differences in brain structure, metabolic maintenance, functional network compensation, or a combination of these and other avenues [3]. Emerging evidence suggests that global brain amyloid PET burden is independently (from entorhinal cortex tau PET burden and cortical thickness) associated with memory decline, but only in the setting of very high amyloid levels present for many years [35]. The association of chromosome 8 rs12056505 with cognitive protection even in the setting of extremely high amyloid burden (global PiB SUVR ≥ 2.0) suggests that its role as a resilience factor cannot be purely ascribed to upstream processes yielding a lesser burden of amyloidosis. We considered other potential explanations for the cognitive resilience observed in rs12056505-C carriers. We did not find evidence that this protective variant was associated with better vascular brain health or with significantly lower tau PET burden in key AD regions. However, the subset of our sample with tau PET data was modest, and it is possible that lower tau accumulation in the setting of amyloidosis could be a mechanism for resilience in this variant that our subsample was underpowered to detect. Non-AD pathophysiology related to TDP-43 [32] or other concomitant degenerative factors can impact cognitive trajectories and could not be directly accounted for in this study in the absence of validated in vivo biomarkers. Alternatively, other mechanisms outside of typical neurodegenerative and vascular disease pathways may be contributing to resilience, such as the immune system and inflammation-related pathways identified in our poly-SNP analyses. A more comprehensive modeling of these putative resilience mechanisms in the context of genetic, sex-related, and lifestyle mediators may facilitate enhanced preventive and therapeutic targeting in AD and related disorders. Specific to this work, Fig. 4 summarizes our overarching experimental design and the discovery of genetic protective factors (and negative testing for other related factors) that were found in the context of resilience to amyloidosis.

Despite these strengths, there are reasons why our main finding should be interpreted with caution. The sample size of our discovery cohort was small in comparison to other GWAS of AD-relevant outcomes, and although the confirmatory association of *APOE* Ɛ4 with resilience in our dataset is reassuring for its broader interpretation, the possibility of a winner’s curse phenomenon regarding our GWAS hit must be acknowledged. Although similar in size and data scope to the MCSA discovery cohort, the ADNI replication sample was also not a perfect match in some characteristics, including differences in selection framework (population-based versus clinical trial sample), amyloid PET tracer, and specific cognitive outcome. In this context, the presence of a validation signal for rs12056505 is encouraging, but is still modest overall and does not rule out the possibility of a false positive. In examining published results from the largest available AD case–control GWAS [36], we cannot find evidence supporting an association of rs12056505 with lower risk of clinically diagnosed probable AD dementia (*p* = 0.90, β = 0.997). Clinical diagnosis of AD dementia is by no means a proxy for resilience to AD pathology, and our data more broadly mirrors conclusions from a recent study on resilience in suggesting that the genetic architectures underlying these outcomes are likely to be meaningfully different [16]. Nevertheless, the lack of clear protective relationship of rs12056505 on clinical AD dementia diagnosis in published data is a limitation. Further, cognitive performance is impacted by numerous factors and as such may not be as specific as fluid- or imaging-based AD-relevant quantitative endophenotypes. In summary, our top discovery is promising but additional validation studies in other cohorts and molecular and functional characterization are needed.

This work has several other limitations. For discovery we leveraged a rich dataset from a population-based sample, which offers the benefit of generalizability to the broader setting of older adults. However, this setting may meaningfully differ from clinical trial samples (including our replication cohort) where a higher proportion of participants have AD biomarker abnormalities and/or cognitive impairment at baseline. An advantage of our study design was that it utilized a well-validated AD biomarker (amyloid PET) and linear regression to model cognitive trajectories, approaches that can be straightforwardly applied to other datasets. However, the optimal approach to modeling resilience is still an active question, and alternative methods based on latent variables (estimating cognitive dysfunction in cross-sectional data over and above that expected from pathology) [16], gene expression data [21], and longitudinal linear mixed models [8] may provide complementary information. In addition, we assessed differential cognitive resilience through the lens of a continuous measure of rate of decline, and acknowledge that our findings may not be applicable to a paradigm whereby cognitive resilience is operationalized as a marker of clinical status (i.e., coping without impairment). We also focused on memory performance as an outcome given its relevance to the setting of clinically typical AD, but recognize that cognitive-genetic associations may well be domain-specific [31] related to the involvement of diverse brain regions and functional networks, and that there may be weaknesses in the use of z-scores for longitudinal studies of cognitive trajectory across individuals who may start from different levels of cognitive ability [11]. Further, we acknowledge that the respective outcome measures for cognitive resilience in the discovery and replication cohorts were not identical, and that future efforts incorporating our study design to additional large samples may benefit from phenotype harmonization to facilitate meta-analysis.

In summary, this study of a population-based sample discovered a novel gene variant and biological pathways associated with cognitive resilience to brain amyloidosis. Additional study of these putative resilience mechanisms may be vital for improved risk stratification and drug targeting for individuals with biomarker-confirmed AD pathophysiology.

## Data Availability

Data from this study is available from the authors upon reasonable request.

## Appendix

Data used in preparation of this article were obtained from the Alzheimer’s Disease Neuroimaging Initiative (ADNI) database (adni.loni.usc.edu). As such, the investigators within the ADNI contributed to the design and implementation of ADNI and/or provided data but did not participate in analysis or writing of this report. A complete listing of ADNI investigators can be found at: http://adni.loni.usc.edu/wp-content/uploads/how_to_apply/ADNI_Acknowledgement_List.pdf.
